# PROSPECTS OF LASSA FEVER CANDIDATE VACCINES

**DOI:** 10.21010/Ajid.v16i2S.6

**Published:** 2022-08-17

**Authors:** Ademusire Babatunde Isaac, Wieczorek Karolina, Alonge Aishat Temitope, Rajen Anuska, Egbe Joanne, Adebambo Deborah, Offorbuike Chiamaka Bianca, Trojan Filip, Przypaśniak Zofia, Oduguwa Ifeoluwa Oluwasegun, Omitoyin Oluwaferanmi, Balogun Toluwalogo Grace

**Affiliations:** 1 College of Medicine, University of Ibadan, Ibadan, Nigeria; 2 Polygeia (Global Health Student Think Tank), Ibadan Branch, Nigeria; 3 Queen Mary University of London Barts and The London School of Medicine and Dentistry, United Kingdom; 4 University College London, Medical School, London, United Kingdom

**Keywords:** Lassa fever, vaccine, innovation, clinical trials

## Abstract

**Background:**

Lassa fever is an acute viral haemorrhagic disease caused by the Lassa virus (LASV). It is endemic in West Africa and infects about 300,000 people each year, leading to approximately 5000 deaths annually. The development of the LASV vaccine has been listed as a priority by the World Health Organization since 2018. Considering the accelerated development and availability of vaccines against COVID-19, we set out to assess the prospects of LASV vaccines and the progress made so far.

**Materials and Methods:**

We reviewed the progress made on twenty-six vaccine candidates listed by Salami *et al*. (2019) and searched for new vaccine candidates through Google Scholar, PubMed, and DOAJ from June to July 2021. We searched the articles published in English using keywords that included “vaccine” AND “Lassa fever” OR “Lassa virus” in the title/abstract.

**Results:**

Thirty-four candidate vaccines were identified – 26 already listed in the review by Salami et al. and an additional 8, which were developed over the last seven years. 30 vaccines are still in the pre-clinical stage while 4 of them are currently undergoing clinical trials. The most promising candidates in 2019 were vesicular stomatitis virus-vectored vaccine and live-attenuated MV/LASV vaccine; both had progressed to clinical trials.

**Conclusions:**

Despite the focus on COVID-19 vaccines since 2020, LASV vaccine is under development and continues to make impressive progress, hence more emphasis should be put into exploring further clinical studies related to the most promising types of vaccines identified.

## Introduction

Lassa fever (LF) is an acute viral hemorrhagic fever caused by the Lassa virus (LASV), a single-stranded RNA virus of the Arenaviridae family. The virus was first isolated in Nigeria in 1969 (World Health Organization, 2021). Rodents, particularly *Mastomys natalensis*, are the virus’s natural hosts. The disease is primarily transmitted to humans through contact with infected rats’ urine or feces (Ilori *et al.*, 2019). Human-to-human infections and laboratory transmission can also occur, particularly in health care settings where infection prevention and control measures are insufficient (World Health Organization, 2021). LF is endemic in the West African countries of Ghana, Benin, Guinea, Liberia, Mali, Sierra Leone, and Nigeria (World Health Organization, 2021); an estimated 300,000 cases occur each year in this region, resulting in 5,000 deaths annually (Ilori *et al.*, 2019). The number of sporadic cases occurring outside of the endemic regions within and outside of Africa is increasing due to an increase in international travel. In the United States, the United Kingdom (Geisbert *et al.*, 2005) and Sweden (Asogun *et al*., 2019), these imported cases have been reported.

Lassa fever has an incubation period of 3–21 days. The early-stage disease is similar to other febrile diseases such as malaria and is usually only suspected after haemorrhagic symptoms develop in the late stages (Keïta *et al.*, 2019). Fever, fatigue, haemorrhage, gastrointestinal symptoms (vomiting, diarrhoea, and stomach ache), respiratory symptoms (cough, chest pain and dyspnea), and neurologic symptoms (disorientation, seizures and unconsciousness) are all common clinical manifestations of the disease. However, cases of LF, especially in endemic areas, may remain asymptomatic (Ilori *et al.*, 2019). In 2018, Nigeria had the highest number of cases of Lassa fever with 171 fatalities reported from the 633 confirmed cases (NCDC, 2018). Currently, there are no vaccines or effective drug therapies for the prevention or treatment of LF. Treatment with intravenous ribavirin has however been shown to reduce mortality and morbidity from LF if started within the first week of onset.

Lassa fever, one of the viruses causing severe haemorrhagic fever in Africa, is a major public health issue in endemic areas. When left untreated, LF can have a case fatality rate of up to 70% (Keïta *et al*., 2019), thus, a preventive vaccine is a critical public health need in endemic areas, particularly to protect health care providers, who are frequently the most at risk of exposure (Geisbert *et al*., 2005), Global efforts are being made to develop new vaccines for the control of diseases of global health importance (Salami *et al*., 2019). Lassa virus (LASV) vaccine development has been identified as a top priority by the World Health Organization (WHO) and the Coalition for Epidemic Preparedness Innovations (CEPI). A WHO Target Product Profile (TPP) of LASV vaccines was developed in June 2017 to aid the development of candidate vaccines for clinical evaluation. The TPP outlined the desired characteristics of potential LASV vaccines in two scenarios: preventive use and reactive/outbreak use. This led to an increase in LASV research, which has facilitated the development of vaccine candidates with pre-clinical proofs of concept that could potentially reduce illness, disease outbreaks, and deaths in humans (Salami *et al*., 2019).

According to the World Health Organization, LF vaccines should be cost-effective and affordable in endemic areas and stable for a reasonable time without extensive cold chain facilities. They should also provide immunity for special populations such as HIV-positive patients, pregnant women, and children. Their efficacy and protection must be at least 3 years; the method of administration should be simple, and a small number of doses should be required to confer long-term immunity (Salami *et al*., 2019). In 2019, Salami *et al* identified about 26 candidate vaccines for LF, some of which met the WHO criteria. Notably, the live-attenuated measles virus/Lassa virus (MV/LASV) platform was found to provide robust protection against LASV in animal models. Vesicular Stomatitis Virus (VSV)-vectored vaccine is another promising live virus vaccine platform, with at least two leading LASV vaccine candidates based on it (Salami *et al*., 2019). The most promising vaccine candidates were reported by (Hallam *et al.*, 2018) to be live attenuated recombinant Mopeia virus/Lassa virus (MOPV/LASV) reassortment and live recombinant VSV and vaccinia vectored vaccines.

Most of the advanced vaccine candidates are expected to undergo human trials soon, though the clinical testing of these products and their deliveries to the most in-need population were reported to present significant problems (Salami *et al*., 2019). In this study, we will follow up on the 26 candidate vaccines identified by Salami and colleagues in 2019 to identify other potential candidate vaccines, discuss their prospects, their rates of development, and highlight factors affecting the development of Lassa fever vaccines.

## Materials and Methods

We reviewed progress made on the 26 vaccine candidates listed by Salami and his colleagues in 2019 through Google Scholar, PubMed, and Directory of Open Access Journals (DOAJ) databases between June 23^rd^, 2021 and July 7^th^, 2021. We also searched for any new vaccine candidates using these databases between July 8^th^, 2021 and July 17^th^, 2021. We searched for articles published in English using keywords that included “vaccine” AND “Lassa Fever” OR “Lassa virus” in the title/abstract.

### Lassa fever vaccines in development

In the review by Salami *et al*. (2019), 26 vaccines currently under development have been reviewed to check the present stage of development, target, and development partners. These are presented, along with progress made since then, in Table 1. This paper reviews the vaccine candidates and checks for progression into potential clinical trials.

**Table 1:** Lassa fever vaccines in development, including progress from 2019 review by Salami et al.

S/N	Vaccine technology	LASV antigen	Stage of development	Development partners	Progression from Salami et al’s 2019 research
*1*	rVSVN4CT1-LASV (VesiculoVaxTM Vesicular Stomatitis Virus Vector) (Salami *et al.*, 2019)		Preclinical	Profectus Biosciences; University of Texas Medical Branch	-
*2*	ML29 L-AttV, rLCMV(IGR/S-S) (Mopiea/ Lassa reassortant) (Salami *et al.*, 2019)	GPC, NP	Preclinical	The Scripps Research Institute, USA	-
*3*	VSV∆G/LASVGPC (VSV vector) (Salami *et al.*, 2019)	GPC	Clinical	International Aids Vaccines Initiative; Public Health Agency of Canada	Phase I clinical trial ongoing. Funding for phase II announced
*4*	RABV-Lassa virus vaccine candidate GPC (Salami *et al.*, 2019)	GPC	Preclinical	National Institute of Allergy and Infectious Diseases (NIAID), National Institute of Health (NIH)	-
*5*	YF 17D GPC (Salami *et al.*, 2019)	GPC	Preclinical	Texas Biomedical Research Institute; University of Louisville; Leiden University Medical Center	-
*6*	ML29 virus – reassortant encodes major immunogenic proteins from LASV and RNA polymerase and Z protein from MOPV (Salami *et al.*, 2019)	GPC+NP	Preclinical	Medigen, Inc. (technology licensed from the University of Maryland); NIAID	-
*7*	Live attenuated rLCMV/CD (Based on Codon Deoptimization) (Salami *et al.*, 2019)	-	Preclinical	The Scripps Research Institute, University of Rochester, USA	-
*8*	GPC441-449 subunit (Salami *et al.*, 2019)	-	Preclinical	Emergent Biosolutions, University of Vermont, California and The Scripps Research Institute	-
*9*	LASV VLP (Salami *et al.*, 2019)	GPC, NP, Z Matrix	Preclinical	Tulane University Health Sciences Center; Autoimmune 10Technologies, LLC; Corgenix Medical Corporation; Vybion, Inc.,; United States Army Medical Research Institute of Infectious Diseases (USAMRIID)	-
*10*	HLA-A02 and 10 HLA-A03-restricted epitopes (Salami *et al.*, 2019)	GPC	Preclinical	The University of Vermont College of Medicine; University of California; Pharmexa-Epimmune	-
*11*	VaxCelerate subunit (Salami *et al.*, 2019)	GP1, GP2	Preclinical	Massachusetts General Hospital; EpiVax, Inc.; 21st Century Biochemicals; University of Washington; MPI Research; Pfenex Inc.	-
*12*	RABV based on chemically inactivated rabies virus containing Lassa Virus coGPC (LASSARAB) ; (Salami *et al.*, 2019)	GPC	Preclinical	Thomas Jefferson University; NIAID; The Geneva Foundation; USAMRIID; IDT Biologika GmbH; Infectious disease research institute (IDRI)	Ready for progress to trials on NHP’s
*13*	PODS Lassa 1; (Salami *et al.*, 2019)	GPC	Preclinical	Cell Guidance Systems; University of Cambridge; Imperial College London; Department of Health - UK	-
*14*	MV-LASV (recombinant measles virus vaccine expressing Lassa virus antigens) (Ramsauer *et al.*, 2015); (Mateo *et al.*, 2019)	GPC + NP, GPC + Z protein	Clinical	Institut Pasteur; Themis Bioscience GmbH	Progressed to clinical trials
*15*	MOPEVAC (Modified Mopeia virus expressing antigens of pathogenic arenaviruses) (Carnec *et al.*, 2018)	GPC	Preclinical	Institut Pasteur	
*16*	Alphavirus replicon encoding LASV genes (Pushko Peter *et al.*, 2001)	GPCwt	Preclinical	Medigen, Inc.; University of Louisville, United States USAMRIID	
*17*	Lassa GPCclamp (molecular clamp technology) (Salami *et al.*, 2019) (Chappell *et al.*, 2021)	GPC	Phase I	The University of Queensland; Australian Government – National Health and Medical Research Council (NHMRC)	Progressed to clinical trials
*18*	ChAdOx1 Lassa (Ewer *et al.*, 2017) (Fischer *et al.*, 2021)		Preclinical	Oxford University	Progressed to trials on NHPs
*19*	MVA Lassa (LassaVac) (Public Health England, n.d.)		Preclinical	Oxford University National Infection Service, Public Health England	
*20*	ChAdOx1-biLAMA (Ewer *et al.*, 2017)		Preclinical	Oxford University/Jansen	
*21*	Viral genome rearrangement for the development of live-attenuated arenavirus vaccines (Cheng *et al.*, 2015)		Preclinical	University of Rochester; The Scripps Research Institute	
*22*	Single cycle infectious viruses as live attenuated arenavirus vaccines (Cheng *et al.*, 2017)		Preclinical	University of Rochester; The Scripps Research Institute	
*23*	Digitally designed Immune Optimized and Selected-Lassa, Ebola, Marburg (in silico design of antigen sequences) (Wagner, 2018)		Preclinical	University of Cambridge; University of Regensberg; Department of Health-UK	
*24*	GEO-LM01 (Labs, n.d.)		Preclinical	GeoVax; The Scripps Research Institute; University of Maryland	Ready to progress to clinical trials
*25*	pLASV-GPC is a DNA plasmid vaccine (Cashman *et al.*, 2017) (Cai, Iwasaki, *et al.*, 2020)		First in human	Inovio Pharmaceuticals; USAMRIID	
*26*	MVA-VLP-TV vaccine (Hemorrhagic Fever Vaccine (Ebola, Sudan, Marburg, Lassa) (Labs, n.d.) (Jiang *et al.*, 2021)	GPC	Preclinical	GeoVax; USAMRIID	Ready to progress to clinical trials

Table 1 (above) summarizes vaccine candidates identified in 2019 and signifies progression, where appropriate. Some of these vaccines are briefly discussed below.

### rVSVN4CT1-LASV (VesiculoVaxTM Vesicular Stomatitis Virus Vector)

Vesicular Stomatitis Virus (VSV) is a virus infecting humans and animals and is an excellent candidate as a pseudovector. Five key proteins are encoded by the genomic RNA: phosphoprotein (P), glycoprotein (G), large protein (L), matrix protein (M), and nucleoprotein (N). Recombinant VSV equipped with a reporter gene instead of a VSV G gene in their genome makes it easier to evaluate infectivity in the study of viral entry, including the identification of viral receptors (Salami *et al*., 2019).

This vaccine has demonstrated usefulness in cynomolgus monkeys in an immune response against filoviruses including Ebola virus (EBOV), Sudan virus (SUDV), and Marburg virus (MARV). Further protection from LASV by a single vaccine is challenging because of several contributing factors. The virus is dissimilar to other species of filoviruses; that is why it has been classified as the *Arenaviridae* family. Overall, 4 groups of LASV have raised concerns about whether one vaccine will provide protection against the whole lineage (Cross *et al*., 2020).

### ML29 L-AttV, rLCMV(IGR/S-S) (Mopiea/ Lassa reassortant)

ML29 is a live-attenuated vaccine (L-AttV) that was shown as a promising candidate in animal studies. Trials demonstrated that a single dose of the vaccine can provide sufficient protection against the virus. Nonetheless, as the ML29 attenuation mechanism is still not entirely understood, researchers have questioned if the vaccine has sufficient phenotypic stability to account for potential future mutations (Cai *et al*., 2020a).

### VSV∆G/LASVGPC (VSV vector)

A phase I clinical trial to evaluate the safety and immunogenicity of rVSV∆G-LASV-GPC vaccine in adults in good general health commenced on June 23, 2021, sponsored by the International AIDS Vaccine Initiative (IAVI).

On June 15, 2021, IAVI was awarded €22.8 million from the European & Developing Countries Clinical Trials Partnership (EDCTP) and the Coalition for Epidemic Preparedness Innovations (CEPI) to conduct Phase IIb clinical trial of a novel vaccine candidate to prevent Lassa fever disease.

IAVI’s rVSV∆G-LASV-GPC uses a recombinant vesicular stomatitis virus (rVSV) vector just like the rVSVN4CT1-LASV vaccine. The same platform was used to produce Merck’s highly efficacious Ebola Zaire virus vaccine, Ervebo®, recently approved by the European Commission, the United States Food and Drug Administration, regulators in several African countries, and prequalified by the World Health Organization (IAVI.ORG, n.d.).

### YF 17D GPC

YF 17D GPC is based on the success of the Yellow Fever 17D (YF17D) vaccine in protecting humans against flaviviruses. The genetic skeleton for this vaccine candidate has been used for the construction of a bivalent YF17D-based recombinant vaccine, which was engineered by combining LASV and YFV (Jiang *et al*., 2021).

This vaccine, however, still does not show any immunogenicity in non-human primates. It is therefore necessary to explain and investigate the causes of low immunogenicity prior to considering it an option for the management of LSV in African regions (Purushotham *et al*., 2019).

### ML29 virus

The reassortant ML29 carrying the L segment from the non-pathogenic Mopeia virus (MOPV) and the S segment from LASV is a vaccine candidate under development. ML29 demonstrated complete protection in validated animal models against a Nigerian strain from clade II, which was responsible for the most severe outbreak recorded in 2018 (Johnson *et al*., 2019).

### LASV VLP

This virus-like particle vaccine is structurally and functionally similar to the LASV virus but lacks any replicative properties; hence, it can be safely recommended for use with low levels of biohazard risk.

In a study by Branco *et al*. (2019) LASV VLP was immunogenic in mice in the absence of adjuvants, with mature IgG responses developing within a few weeks of the first immunization. These findings highlight the importance of using a VLP platform to develop the best vaccine candidates for Lassa hemorrhagic fever and they call for more research in lethal challenge animal models to establish their protective potential.

### HLA-A02 and 10 HLA-A03-restricted epitopes

This vaccine is an attempt to develop one vaccine for the 7 strains of arenaviruses namely: Lymphocytic choriomeningitis virus (LCMV), Guanarito virus (GTOV), Junin virus (JUNV), Machupo virus (MACV), Sabia virus (SABV), Lassa and Whitewater Arroyo viruses. This can be achieved by choosing a molecule (epitope) common to them and targeting shared structural parts. HLA-transgenic mice were challenged following the peptide pool immunization and the magnitude of epitope-specific CD8+ T cell response was sufficient to reduce the viral titers. However, it was less extensive when contrasted with the responses found after individual peptide immunization (Salami *et al*., 2019).

### LASSARAB

This vaccine has been designed to protect from both Lassa fever and rabies and was promising in the initial stages of testing. The candidate is based on a weakened, inactivated rabies vector. New studies indicate that LASSARAB injected with a GLA-SE adjuvant causes robust antibody production against both Lassa fever and rabies in guinea pigs and mouse experiments. Animals were protected from disease up to 58 days after administration (Abreu-Mota *et al*., 2018).

Previous research shows that immune responses are not usually proportionally correlated with protection from LSV. New studies, however, showed that high numbers of IgG antibodies which attach to the LSV proteins do in fact correlate with the protection from the virus. Thus, the levels of this antibody have the potential to be used to determine the usefulness and efficacy of the vaccine. The next step is to evaluate the experimental vaccine in nonhuman primates before advancing to human clinical trials (Abreu-Mota *et al*., 2018).

### pLASV-GPC

DNA plasmid vaccination involves plasmid expressing gene(s) encoding the antigen(s) being introduced to cells, which stimulate an immune response (Cashman *et al*., 2017). pLASV-GPC encodes the GPC gene of the LF (Josiah strain), and when administered in 3 doses dermally, it protected guinea pigs from LF Virus-associated illness and death. The result was replicated in NHPs. Initially, this has been the most promising vaccine candidate as it is the first to commence human trials (Salami *et al*., 2019).

### MV-LASV

The MV-LASV vaccine candidate is a recombinant live attenuated measles vectored vaccine, presenting antigen of the foreign pathogen which has progressed to clinical trials since Salami, *et al*’s (2019) research. It offered strong protection in animal models demonstrating that viral replication and stimulation of innate immune responses are fundamental for an effective adaptive immune response in Lassa fever and has formed the basis for previously licensed vaccines (Henao-Restrepo *et al*., 2015).

### MOPEVAC

Researchers have successfully inoculated NHPs against LF Virus using a modified Mopeia virus (MOPEVAC) (Carnec *et al*., 2018). Exoribonuclease (ExoN) activity in NP of MOPV results in the multiplication of a hyper attenuated strain (MOPV_ExoN6b_) which promotes a strong immune response. The vaccine may result in post-exposure prophylaxis evidenced in previous trials by the 100% survival rate in guinea pigs vaccinated 48h after infection (Zapata *et al*., 2013).

### VLPs

GEO-LM01 is based on the Modified Vaccinia Ankara (MVA) Virus-Like Particle (VLP) platform (*GEO-LM01 Lassa Fever Vaccine*, 2021). It generates non-infectious VLPs containing viral surface proteins which can elicit strong T cell and B cell immune responses, which has allowed VLP-based vaccines to be developed for hepatitis B virus and human papillomavirus (Branco *et al*., 2010). According to Geovax, GEO-LM01 has been validated preclinically and is ready for phase I clinical trials. Vaccination of laboratory mice with LF Virus VLPs (3 doses) via the MVA-VLP-TV vaccine resulted in robust immune responses (Branco *et al*., 2010; Labs, n.d.). According to GeoVax, the MVA-VLP-TV vaccine has been validated preclinically and is ready for phase I clinical trials.

ChAdOx1 Lassa and ChAdOx1-biLAMA circumvent pre-existing immunity to human adenoviruses (Branco *et al*., 2010). Existing ChAdOx vaccines such as the ChAdOx1 nCoV-19 Covid-19 Vaccine which shows 70·4% efficacy against Covid-19 highlight the likely success of this blueprint for vaccines (Voysey *et al*., 2021).

### Alphavirus replicon encoding LASV genes

The alphavirus replicon encoding Lassa Fever Virus genes is the only vaccine known to protect NHPs against at least three natural genotypes of Lassa virus (Lukashevich & Pushko, 2016).

### Lassa GPCclamp

The Lassa GPCclamp-based vaccine has completed phase I clinical trials with 99% of vaccinated participants producing a neutralizing immune response. However, it has not progressed to phase II trials due to cross-reactivity in HIV patients.

### Other Lassa fever vaccine candidates

Salami *et al*. (2019) did not include a few vaccine candidates in their 2019 review. Also, new vaccine candidates have emerged since 2019; their stages of development are reported in the table below.

### LHF-535

A small-molecule viral entry inhibitor was developed to protect against the Lassa virus by targeting the viral envelope glycoprotein. It is currently the only viral entry inhibitor vaccine for the Lassa virus. Since its development, the phase I clinical trial has been completed and reported to be well tolerated (Health Newswire, 2021; Madu *et al*., 2018).

### MVALassaNP

The MVALassaNP was found to trigger both humoral and cell-mediated immunity against the Lassa virus. It was proposed to be protective against the Lassa virus due to its cell-mediated function and also halted disease progression in pigs (Kennedy *et al*., 2019).

### Lassa VRPs

The vaccine combines the benefits of a live vaccine with that of a single cycle of replication. It demonstrated protective immunity against the disease in lethal guinea pig models (Kainulainen *et al*., 2018).

### MeV-NP

MeV-NP was developed by generating MeV expressing NP protein. It induced efficient protection in a single dose in monkeys (Mateo *et al*., 2019).

### rLASV-GPC/CD LAV

Researchers from the University of Rochester and Schipps Research Institute adopted a model used to develop a recombinant form of the mammarenavirus lymphocytic choriomeningitis virus (rLCMV) using a colon deoptimization glycoprotein (CD GPC ). They incorporated the CD-GPC to develop rLASV-GPC/CD (Cai *et al*., 2020b). In low single doses, the vaccine offered complete protection to Hartley guinea pigs (Cai *et al*., 2020a). Currently, the vaccine is funded by the Coalition for Epidemic Preparedness Innovations (CEPI) and is still in the preclinical phase.

### ChAdOx1-Lassa-GPC

This vaccine comprises of proprietary chimpanzee adenovirus vector platform (ChAdOx1) expressing the Josiah strain LASV GPC: ChAdOX1- Lassa-GP, this vector is derived from chimpanzee adenovirus Y25 (Purushotham *et al*., 2019). ChAdOx1-vectored Lassa fever vaccine encodes the full-length Josiah strain LASV GPC sequence (ChAdOx1-Lassa-GPC) and protective efficacy was seen after one single dose was given to guinea pigs (Fischer *et al*., 2021). The vaccine was developed by the University of Oxford and Janssen Vaccines & Prevention B.V. and is being funded by CEPI (Purushotham *et al*., 2019).

### rVSV- N4ΔG -LASVGPC in Quadrivalent VesiculoVax

The vaccine was designed for Lassa virus (LASV), Ebola Virus (EBOV), Sudan Ebola virus (SUDV) and Marburg Virus (MARV) (Cross *et al*., 2020). It encodes the Josiah strain glycoprotein and for the LASV it completely lacks Vesicular Stomatitis Virus (VSV) G protein hence it has been called N4ΔG (Cross *et al*., 2020).

### IN0-4500(DNA)

IN0-4500 was developed by Inovio’s Pharmaceutical and encodes the LASV Josiah strain, GPC gene, funded by CEPI. It was demonstrated to offer 100% efficacy after two immunizations at four sites (Purushotham *et al.*, 2019). It is still in the preclinical phase of development (Bernasconi *et al*., 2020).

## Discussion

There is substantial progress in the development of LF vaccines using a variety of novel technologies. In the review by Salami *et al*. (2019), twenty-six vaccine candidates were identified in preclinical stages. Since then, three of them have progressed to clinical trials, with one securing funding for Phase 2 trials. Most vaccine candidates have significantly progressed since 2019 with only a few being withdrawn. Out of eight new candidates included in Table 2., one vaccine candidate has progressed to clinical Phase 1 trial.

**Table 2:** New Lassa fever Vaccine candidates not included in the review of 2019 by Salami *et al*.

*S/N*	*Name of Vaccine*	*Lasv antigen*	*Clinical trial stage*	*Funding organization*	*Vaccine development progression*	*Year of Development*
*1*	LHF-535 (Health Newswire, 2021; Madu *et al.*, 2018)	GP2	Clinical	National Institute of Allergy and Infectious Disease, Wellcome Trust Translation Fund Award	Successful clinical trial result reported March 2021	2016
*2*	MVALassaNP (Kennedy *et al.*, 2019)	NP	Preclinical	Official Development Assistance (ODA)	-	2019
*3*	Lassa VRPs (Kainulainen *et al.*, 2018)	GPC	Preclinical	Centre for Disease Control and Prevention	-	2019
*4*	MeV-NP (Mateo *et al.*, 2019)	NP	Preclinical	Fondation pour l’innovation en infectiologie (FINOVI)	-	2019
*5*	rLASV-GPC/CD LAV (Ibukun, 2020)	GP & NP	Preclinical	Coalition for Epidemiological Preparedness Innovation (CEPI)	-	2019
*6*	ChAdOx1-Lassa-GPC (Fischer *et al.*, 2021)		Preclinical	CEPI	-	2020
*7*	rVSV-N4DG-LASVGPC in Quadrivalent VesiculoVax (Cross *et al.*, 2020)	GP	Preclinical	NIADH (National Institute of Allergy and Infectious Disease), National Human Genome Research Institute(NHGRI), ACEGID and World Bank	-	2014
*8*	IN0-4500(DNA) (Purushotham *et al.*, 2019)	GP	Preclinical	CEPI	-	2013

The most promising candidates in 2019 were Vesicular Stomatitis Virus (VSV)-vectored vaccine and Live-attenuated MV/LASV have already progressed to clinical trials. Other candidates that have seen progress include Mopeia, Lassa Virus reassortants with various antigens as well as DNA platforms. The review of new candidates has shown that additional eight vaccines have been designed, seven of them currently in preclinical stages. Some of these are already being evaluated for clinical phases due to successful trials on animal subjects. We, therefore, recommend that further studies be tailored around the four types which have successfully progressed to clinical trials.

## Conclusions

To be used in endemic regions, the vaccine must be cost-effective, affordable, and sustainable according to the WHO’s target product profiles (World Health Organization, 2019). The vaccine development has been described as crucial by the WHO since LF was identified as a potential risk for a pandemic. Following the problems in vaccine production, particularly that posed by LSV lineage diversity, there has been an increased demand to see clinical candidates for vaccines that can safely target multiple strains of the virus. It is critical that all vaccines based on the LASV Josiah strain are tested across all lineages to ensure universality. To conclude, since 2019 and despite the focus on the development of COVID-19 vaccines globally, some of the Lassa virus vaccine candidates have significantly progressed in both preclinical and clinical tests shaping a profoundly mature pipeline in terms of safety and usefulness.

### Conflict of interest

The authors have identified no conflict of interest in this study.

**Abbreviations:**
CD8+ T cell: Cluster of Differentiation 8-positive T Lymphocytes;
CEPI: Coalition for Epidemic Preparedness Innovations;
ChAdOx1: Chimpanzee Adenovirus vector platform;
COVID-19: Coronavirus Disease 2019
DNA: Deoxyribonucleic acid;
DOAJ: Directory of Open Access Journals;
EBOV: Ebola virus;
G: Glycoprotein
GEO-LM01: GeoVax Labs Lassa fever vaccine;
GLA-SE: Glucopyranosyl lipid adjuvant formulated in a squalene-in-water emulsion;
GP1: Glycoprotein 1;
GP2: Glycoprotein 2;
GPC: Glycoprotein complex;
GPCwt: Wild type Glycoprotein;
GTOV: Guanarito virus;
HIV: Human Immunodeficiency Virus;
HLA: Human leukocyte antigens
IDRI: Infectious Disease Research Institute;
IgG: Immunoglobulin G;
JUNV: Junin virus;
L: Large protein;
L-AttV: Live-attenuated vaccine;
LASSARAB: Lassa Fever Rabies Virus Vaccine;
LASV: Lassa Virus; LASV VLP Lassa Virus Virus-Like Particle;
LCMV: Lymphocytic Choriomeningitis Virus;
LF: Lassa Fever;
LSV: Lily Symptomless Virus;
M: Matrix protein;
MACV: Machupo virus:
MARV: Marburg virus;
ML29: Mopeia/Lassa reassortant;
MOPEVAC: Modified Mopeia virus expressing antigens of pathogenic arenaviruses
MOPV/LASV: Mopeia virus/Lassa virus;
MVA: Modified Vaccinia Ankara;
MVALassaNP: Modified Vaccinia Ankara expressing the nucleoprotein from Lassa virus;
MV/LASV: Measles virus/Lassa virus;
MV-LASV: Recombinant measles virus vaccine expressing Lassa virus antigens;
N: Nucleoprotein;
NCDC: Nigeria Centre for Disease Control
NIAID: National Institute of Allergy and Infectious Diseases;
NIH: National Institute of Health;
NHPs: Non-Human Primate Models;
NP: Nucleoprotein;
P: Phosphoprotein; pLASV-GPCLassa virus glycoprotein precursor gene
PODS: Polyhedra Delivery System; PubMedPublic Medline;
RABV: Rabies Virus;
rLCMV/CD: Recombinant Lymphocytic Choriomeningitis Virus Based on Codon Deoptimization;
RNA: Ribonucleic acid;
SABV: Sabia virus
SUDV: Sudan virus;
TPP: Target Product Profile;
UK: United Kingdom;
USAMRIID: United States Army Medical Research Institute of Infectious Diseases;
VLP: Virus-Like Particle;
VSV: Vesicular Stomatitis Virus;
WHO: World Health Organization;
YF 17D: Yellow Fever 17D

## References

[1] 2018 Lassa Fever Outbreak in Nigeria:NCDC Situation Report #52 - 31 December 2018 - Nigeria. (n.d.).

[2] Abreu-Mota T, Hagen K. R, Cooper K, Jahrling P. B, Tan G, Wirblich C, Johnson R. F, Schnell M. J (2018). Non-neutralizing antibodies elicited by recombinant Lassa-Rabies vaccine are critical for protection against Lassa fever. Nature Communications.

[3] Asogun D. A, Günther S, Akpede G. O, Ihekweazu C, Zumla A (2019). Lassa Fever:Epidemiology, Clinical Features, Diagnosis, Management and Prevention. Infectious Disease Clinics of North America.

[4] A Trial to Evaluate the Optimal Dose of MV-LASV (V182-001). (n.d.).

[5] Bernasconi V, Kristiansen P. A, Whelan M, Román R. G, Bettis A, Yimer S. A, Gurry C, Andersen S. R, Yeskey D, Mandi H, Kumar A, Holst J, Clark C, Cramer J. P, Røttingen J.-A, Hatchett R, Saville M, Norheim G (2020). Developing vaccines against epidemic-prone emerging infectious diseases. Bundesgesundheitsblatt, Gesundheitsforschung, Gesundheitsschutz.

[6] Branco L. M, Grove J. N, Geske F. J, Boisen M. L, Muncy I. J, Magliato S. A, Henderson L. A, Schoepp R. J, Cashman K. A, Hensley L. E, Garry R. F (2010). Lassa virus-like particles displaying all major immunological determinants as a vaccine candidate for Lassa hemorrhagic fever. Virology Journal.

[7] Cai Y, Iwasaki M, Motooka D, Liu D. X, Yu S, Cooper K, Hart R, Adams R, Burdette T, Postnikova E. N, Kurtz J, St Claire M, Ye C, Kuhn J. H, Martínez-Sobrido L, de la Torre J. C (2020b). A Lassa Virus Live-Attenuated Vaccine Candidate Based on Rearrangement of the Intergenic Region. mBio.

[8] Cai Y, Ye C, Cheng B, Nogales A, Iwasaki M, Yu S, Cooper K, Liu D. X, Hart R, Adams R, Brady T, Postnikova E. N, Kurtz J, St Claire M, Kuhn J. H, de la Torre J. C, Martínez-Sobrido L (2020a). A Lassa Fever Live-Attenuated Vaccine Based on Codon Deoptimization of the Viral Glycoprotein Gene. mBio.

[9] Carnec X, Mateo M, Page A, Reynard S, Hortion J, Picard C, Yekwa E, Barrot L, Barron S, Vallve A, Raoul H, Carbonnelle C, Ferron F, Baize S (2018). A Vaccine Platform against Arenaviruses Based on a Recombinant Hyperattenuated Mopeia Virus Expressing Heterologous Glycoproteins. Journal of Virology.

[10] Cashman K. A, Wilkinson E. R, Shaia C. I, Facemire P. R, Bell T. M, Bearss J. J, Shamblin J. D, Wollen S. E, Broderick K. E, Sardesai N. Y, Schmaljohn C. S (2017). A DNA vaccine delivered by dermal electroporation fully protects cynomolgus macaques against Lassa fever. Human Vaccines &Immunotherapeutics.

[11] Chappell K. J, Mordant F. L, Li Z, Wijesundara D. K, Ellenberg P, Lackenby J. A, Cheung S. T. M, Modhiran N, Avumegah M. S, Henderson C. L, Hoger K, Griffin P, Bennet J, Hensen L, Zhang W, Nguyen T. H. O, Marrero-Hernandez S, Selva K. J, Chung A. W, …Munro T. P (2021). Safety and immunogenicity of an MF59-adjuvanted spike glycoprotein-clamp vaccine for SARS-CoV-2:a randomised, double-blind, placebo-controlled, phase 1 trial. The Lancet Infectious Diseases.

[12] Cheng B. Y. H, Nogales A, de la Torre J. C, Martínez-Sobrido L (2017). Development of live-attenuated arenavirus vaccines based on codon deoptimization of the viral glycoprotein. Virology.

[13] Cheng B. Y. H, Ortiz-Riaño E, de la Torre J. C, Martínez-Sobrido L (2015). Arenavirus genome rearrangement for the development of live attenuated vaccines. Journal of Virology.

[14] Cross R. W, Xu R, Matassov D, Hamm S, Latham T. E, Gerardi C. S, Nowak R. M, Geisbert J. B, Ota-Setlik A, Agans K. N, Luckay A, Witko S. E, Soukieh L, Deer D. J, Mire C. E, Feldmann H, Happi C, Fenton K. A, Eldridge J. H, Geisbert T. W (2020). Quadrivalent VesiculoVax vaccine protects nonhuman primates from viral-induced hemorrhagic fever and death. The Journal of Clinical Investigation.

[15] Ewer K, Sebastian S, Spencer A. J, Gilbert S, Hill A. V. S, Lambe T (2017). Chimpanzee adenoviral vectors as vaccines for outbreak pathogens. Human Vaccines &Immunotherapeutics.

[16] Fischer R. J, Purushotham J. N, van Doremalen N, Sebastian S, Meade-White K, Cordova K, Letko M, Jeremiah Matson M, Feldmann F, Haddock E, LaCasse R, Saturday G, Lambe T, Gilbert S. C, Munster V. J (2021). ChAdOx1-vectored Lassa fever vaccine elicits a robust cellular and humoral immune response and protects guinea pigs against lethal Lassa virus challenge. NPJ Vaccines.

[17] Geisbert T. W, Jones S, Fritz E. A, Shurtleff A. C, Geisbert J. B, Liebscher R, Grolla A, Ströher U, Fernando L, Daddario K. M, Guttieri M. C, Mothé B. R, Larsen T, Hensley L. E, Jahrling P. B, Feldmann H (2005). Development of a new vaccine for the prevention of Lassa fever. PLoS Medicine.

[18] GEO-LM01 Lassa Fever Vaccine. (n.d.).

[19] Hallam H. J, Hallam S, Rodriguez S. E, Barrett A. D. T, Beasley D. W. C, Chua A, Ksiazek T. G, Milligan G. N, Sathiyamoorthy V, Reece L. M (2018). Baseline mapping of Lassa fever virology, epidemiology and vaccine research and development. NPJ Vaccines.

[20] Health Newswire. (2021, March 24) Kineta Presents Clinical Study Results of LHF-535 at the International Conference on Antiviral Research (ICAR).

[21] Henao-Restrepo A. M, Longini I. M, Egger M, Dean N. E, Edmunds W. J, Camacho A, Carroll M. W, Doumbia M, Draguez B, Duraffour S, Enwere G, Grais R, Gunther S, Hossmann S, Kondé M. K, Kone S, Kuisma E, Levine M. M, Mandal S, …Røttingen J.-A (2015). Efficacy and effectiveness of an rVSV-vectored vaccine expressing Ebola surface glycoprotein:interim results from the Guinea ring vaccination cluster-randomised trial. The Lancet.

[22] IAVI.ORG. (n.d.) EDCTP and CEPI funding moves IAVI's Lassa fever vaccine candidate into advanced clinical development.

[23] Ibukun F. I (2020). Inter-Lineage Variation of Lassa Virus Glycoprotein Epitopes:A Challenge to Lassa Virus Vaccine Development. Viruses.

[24] Ilori E. A, Furuse Y, Ipadeola O. B, Dan-Nwafor C. C, Abubakar A, Womi-Eteng O. E, Ogbaini-Emovon E, Okogbenin S, Unigwe U, Ogah E, Ayodeji O, Abejegah C, Liasu A. A, Musa E. O, Woldetsadik S. F, Lasuba C. L. P, Alemu W, Ihekweazu C, Nigeria Lassa Fever National Response Team (2019). Epidemiologic and Clinical Features of Lassa Fever Outbreak in Nigeria, January 1-May 6, 2018. Emerging Infectious Diseases.

[25] Jiang J, Ramos S. J, Bangalore P, Elwood D, Cashman K. A, Kudchodkar S. B, Schultheis K, Pugh H, Walters J, Tur J, Yan J, Patel A, Muthumani K, Schmaljohn C. S, Weiner D. B, Humeau L. M, Broderick K. E (2021). Multivalent DNA Vaccines as A Strategy to Combat Multiple Concurrent Epidemics:Mosquito-Borne and Hemorrhagic Fever Viruses. Viruses.

[26] Johnson D. M, Jokinen J. D, Lukashevich I. S (2019). Attenuated Replication of Lassa Virus Vaccine Candidate ML29 in STAT-1-/- Mice. Pathogens.

[27] Kainulainen M. H, Spengler J. R, Welch S. R, Coleman-McCray J. D, Harmon J. R, Klena J. D, Nichol S. T, Albariño C. G, Spiropoulou C. F (2018). Use of a Scalable Replicon-Particle Vaccine to Protect Against Lethal Lassa Virus Infection in the Guinea Pig Model. The Journal of Infectious Diseases.

[28] Keïta M, Kizerbo G. A, Subissi L, Traoré F. A, Doré A, Camara M. F, Barry A, Pallawo R, Baldé M. O, Magassouba N, Djingarey M. H, Fall I. S (2019). Investigation of a cross-border case of Lassa fever in West Africa. BMC Infectious Diseases.

[29] Kennedy E. M, Dowall S. D, Salguero F. J, Yeates P, Aram M, Hewson R (2019). A vaccine based on recombinant modified Vaccinia Ankara containing the nucleoprotein from Lassa virus protects against disease progression in a guinea pig model. Vaccine.

[30] Labs, G. V. (n.d.) GeoVax Labs, Inc Factsheet.

[31] Lukashevich I. S, Pushko P (2016). Vaccine platforms to control Lassa fever. Expert Review of Vaccines.

[32] Madu I. G, Files M, Gharaibeh D. N, Moore A. L, Jung K.-H, Gowen B. B, Dai D, Jones K. F, Tyavanagimatt S. R, Burgeson J. R, Korth M. J, Bedard K. M, Iadonato S. P, Amberg S. M (2018). A potent Lassa virus antiviral targets an arenavirus virulence determinant. PLoS Pathogens.

[33] Mateo M, Reynard S, Carnec X, Journeaux A, Baillet N, Schaeffer J, Picard C, Legras-Lachuer C, Allan R, Perthame E, Hillion K.-H, Pietrosemoli N, Dillies M.-A, Barrot L, Vallve A, Barron S, Fellmann L, Gaillard J.-C, Armengaud J, …Baize S (2019). Vaccines inducing immunity to Lassa virus glycoprotein and nucleoprotein protect macaques after a single shot. Science Translational Medicine.

[34] Public Health England. (n.d.) LassaVacc. UK Reserach and Innovation.

[35] Purushotham J, Lambe T, Gilbert S. C (2019). Vaccine platforms for the prevention of Lassa fever. Immunology Letters.

[36] Pushko Peter, Geisbert Joan, Parker Michael, Jahrling Peter, and Smith Jonathan (2001). Individual and Bivalent Vaccines Based on Alphavirus Replicons Protect Guinea Pigs against Infection with Lassa and Ebola Viruses. Journal of Virology.

[37] Ramsauer K, Schwameis M, Firbas C, Müllner M, Putnak R. J, Thomas S. J, Desprès P, Tauber E, Jilma B, Tangy F (2015). Immunogenicity, safety, and tolerability of a recombinant measles-virus-based chikungunya vaccine:a randomised, double-blind, placebo-controlled, active-comparator, first-in-man trial. The Lancet Infectious Diseases.

[38] Salami K, Gouglas D, Schmaljohn C, Saville M, Tornieporth N (2019). A review of Lassa fever vaccine candidates. Current Opinion in Virology.

[39] Voysey M, Clemens S. A. C, Madhi S. A, Weckx L. Y, Folegatti P. M, Aley P. K, Angus B, Baillie V. L, Barnabas S. L, Bhorat Q. E, Bibi S, Briner C, Cicconi P, Collins A. M, Colin-Jones R, Cutland C. L, Darton T. C, Dheda K, Duncan C. J. A, …Oxford COVID Vaccine Trial Group (2021). Safety and efficacy of the ChAdOx1 nCoV-19 vaccine (AZD1222) against SARS-CoV-2:an interim analysis of four randomised controlled trials in Brazil, South Africa, and the UK. The Lancet.

[40] Wagner R, (2018, August 29) Trivalent Lassa, Ebola and Marburg viral vaccine (Tri-LEMvac). University of Regensburg Institute of Medical Microbiology and Hygiene Molecular Microbiology (Virology).

[41] World Health Organization (2019). Lassa Fever Research and Development Roadmap. Lassa Fever - Advanced Draft.

[42] World Health Organization (2021) Lassa Fever.

[43] Zapata J. C, Poonia B, Bryant J, Davis H, Ateh E, George L, Crasta O, Zhang Y, Slezak T, Jaing C, Pauza C. D, Goicochea M, Moshkoff D, Lukashevich I. S, Salvato M. S (2013). An attenuated Lassa vaccine in SIV-infected rhesus macaques does not persist or cause arenavirus disease but does elicit Lassa virus-specific immunity. Virology Journal.

